# Molecular Structure Discovery for Untargeted Metabolomics Using Biotransformation Rules and Global Molecular Networking

**DOI:** 10.1021/acs.analchem.4c01565

**Published:** 2025-02-04

**Authors:** Margaret R. Martin, Wout Bittremieux, Soha Hassoun

**Affiliations:** † Department of Computer Science, Tufts University, Medford, Massachusetts 02155, United States; ‡ Department of Computer Science, University of Antwerp, 2020 Antwerp, Belgium; § Department of Chemical and Biological Engineering, Tufts University, Medford, Massachusetts 02155, United States

## Abstract

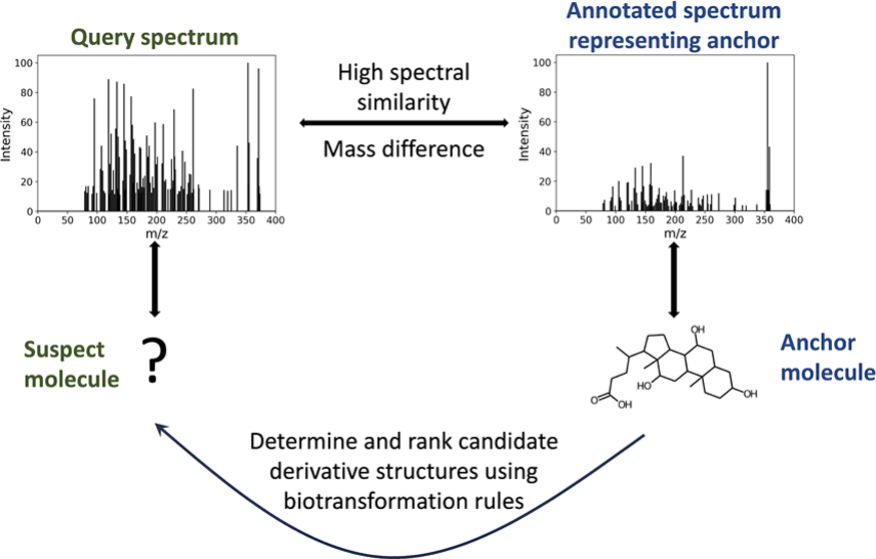

Although untargeted mass spectrometry-based metabolomics is crucial for understanding life’s molecular underpinnings, its effectiveness is hampered by low annotation rates of the generated tandem mass spectra. To address this issue, we introduce a novel data-driven approach, Biotransformation-based Annotation Method (BAM), that leverages molecular structural similarities inherent in biochemical reactions. BAM operates by applying biotransformation rules to known “anchor” molecules, which exhibit high spectral similarity to unknown spectra, thereby hypothesizing and ranking potential structures for the corresponding “suspect” molecule. BAM’s effectiveness is demonstrated by its success in annotating query spectra in a global molecular network comprising hundreds of millions of spectra. BAM was able to assign correct molecular structures to 24.2% of examined anchor-suspect cases, thereby demonstrating remarkable advancement in metabolite annotation.

## Introduction

Metabolomics, empowered by untargeted mass spectrometry (MS), is pivotal in advancing phenotyping and biomarker discovery by profiling thousands of small molecules using tandem mass spectrometry (MS/MS). However, a critical challenge hampers its full potential: the low MS/MS spectrum annotation rate. Despite technological advancements and expanding spectral libraries, a significant portion of spectra remain unidentified. For example, only a small minority of spectra in key public data repositories like Global Natural Product Social Molecular Networking (GNPS)/MassIVE^1^ has been successfully annotated.^2^

This gap in annotation is particularly pressing given the crucial role of metabolites as direct products of metabolic processes. Some methods attempt to address this by leveraging the biochemical transformations and structural similarities intrinsic to metabolites.^3,4^ These approaches utilize MS/MS spectral similarity relationships to predict structural relationships and integrate reactants from metabolic networks to improve annotation confidence, as shown through experimental validation. These works predict structural relationships through various methods, such as extending known metabolic networks,^5^ utilizing metabolic networks, spectral similarity networks, and global peak correlation networks,^6^ and extending molecular networks.^7^ However, despite these efforts, existing methodologies fall short in effectively interpreting the vast majority of MS/MS data produced in untargeted metabolomics experiments. This limitation not only restricts biological insights that could be gleaned from expensive and critical studies, but also hinders a deeper understanding of metabolism and its products. Thus, there is an urgent need for innovative approaches that can bridge this gap in MS/MS spectrum annotation, enabling more comprehensive exploration and interpretation of the metabolome in untargeted metabolomics studies.

To address this problem, we have developed the Biotransformation-based Annotation Method (BAM). At its core, BAM leverages the principle that reactant and products of biochemical reactions often share molecular substructures, leading to high spectral similarity.^8^ This spectral similarity, when analyzed within a biological context, provides a more biologically relevant and comprehensive approach to annotation compared to traditional methods that rely solely on spectral similarity. BAM operates by identifying and applying biotransformation rules to an “anchor” molecule—a known compound whose MS/MS spectrum exhibits high similarity to a given query spectrum. These rules, drawn from extensive biochemical databases like RetroRules^9^ and KEGG,^10^ encapsulate the promiscuous behavior of enzymes toward substrates. Specifically, they include a molecular substructure specifying a reaction center and the structural changes occurring therein. BAM systematically employs these rules to generate and rank potential molecular structures (“derivatives”) for the “suspect” molecule—the unknown compound associated with the query spectrum. To do so, BAM makes use of PROXIMAL2^11^ to apply the biotransformation rules to the anchor molecule, identifying suitable reaction centers for the proposed structural changes. Next, the graph neural network-based GNN-SOM tool is used to predict the site-of-metabolism and rank these putative derivatives based on the likelihood of each atom being a reaction center.^12^

## Methods

**Figure 1:**
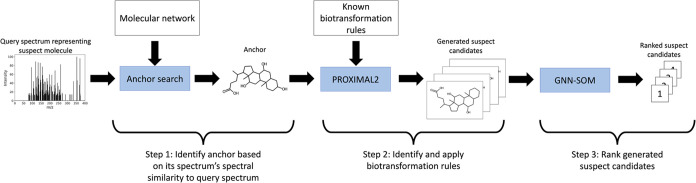
BAM overview. BAM generates and ranks candidate structures for the suspect molecule the unknown compound associated with the query spectrum through three steps. For each query spectrum representing a suspect, an anchor, which is a known molecule with its labeled spectrum, is first determined based on spectral similarity. There are a number of methods to perform anchor search. Here, we use a precomputed molecular network, that we refer to as the global molecular network, to efficiently identify anchor-suspect pairs that exhibit high spectral similarity. Next, potential biotransformation rules, represented by reactant-product pairs, are identified based on the mass difference between the anchor and the suspect and are applied to the anchor structure to generate structural candidates for the suspect structure using PROXIMAL2. Then, the generated candidates are ranked using GNN-SOM.

BAM consists of three steps (Figure 1):

1. For a given query spectrum, determine an annotated spectrum associated with an anchor molecule based on high spectral similarity.
2. Apply relevant biotransformation rules that match the observed mass difference between the anchor and suspect to generate candidate structures—derivatives—for the query spectrum.
3. Rank the derivatives based on the likelihoods of the applied biotransformation.

Next, we will describe each of these steps in detail.

### Identification of Anchor Molecules Based on Spectral Similarity

In the initial step, for a given query MS/MS spectrum, BAM identifies an annotated spectrum associated with an anchor molecule that closely resembles the query spectrum in terms of spectral similarity, a process which we conceptually refer to as “anchor search” (Figure 1). In general, anchor search can be achieved through various methods. “Identity” search compares a query spectrum against annotated spectra in a spectral library to transfer a compound label when a high-scoring match is achieved.^13^ However, as an anchor–suspect pair consists of two spectra that correspond to structurally related yet nonidentical molecules, “identity” spectral library search is not appropriate to use with BAM. “Analog” search refers to library searching using a wide precursor mass tolerance to find matches to spectra of structurally related molecules. For example, analog spectral library search using modified cosine similarity yields such molecule pairs through partial MS/MS spectrum matching while accounting for shifted fragment ions.^14^ However, by using a wide precursor mass tolerance, a massive search space needs to be considered for each query spectrum. Such a search at a repository scale is computationally expensive.^15,16^ As a more efficient alternative, we utilized a precomputed molecular network, which we refer to as the global molecular network.^15^

Molecular networking uses spectral alignment via modified cosine similarity to visualize spectra and any corresponding annotations as a network, which enables additional interpretation of query spectra.^17^ The global molecular network was recently constructed from 521 million MS/MS spectra in 1335 public projects deposited to the GNPS/MassIVE,^1^ MetaboLights,^18^ and Metabolomics Workbench^19^ public data repositories. As is standard methodology in molecular networking, the nodes of the global molecular network were annotated using standard spectral library searching against the default GNPS spectral libraries, which resulted in 454,091 annotated nodes, and thus, we were able to obtain promising structural relationships efficiently. From these nodes, we identified paired annotated compounds with high spectral similarity (modified cosine similarity above 0.8). While the majority of the edges in the molecular network (8,599,249) connect pairs of unannotated nodes, only a small number of edges (616,602) connect pairs of annotated nodes. Furthermore, 30,184 edges in the global molecular network connect pairs of nodes that represent structurally unique molecules. Consequently, whereas analog spectral library searching provides similar results to those obtained via molecular networking, instead, we reused the existing global molecular network to efficiently generate anchor–suspect pairs from millions of MS/MS spectra based on previously computed results.

### Applying Relevant Biotransformation Rules

Once an anchor molecule is identified, BAM applies relevant biotransformation rules. These rules can be sourced from databases like RetroRules,^9^ KEGG,^10^ or Metacyc.^20^ In this case, RetroRules was used due to its sizable collection of biotransformation rules. To extract and apply these rules, we employed PROXIMAL2.^11^ This tool aligns each reactant–product pair using a maximum common substructure algorithm to identify the reaction center, and then creates a lookup table entry defining the changes at the reaction center and its neighbors. These entries, encoded using KEGG atom types,^21^ are generated for all reactant–product pairs. Finally, for a given anchor–suspect pair, BAM applies the rules whose mass difference matches the observed precursor mass difference between the anchor and suspect to generate candidate derivatives for the query molecule. We evaluated candidate generation of BAM by reporting the recall. Here, we define recall as the percentage of queries that the method generates the true structure of the suspect. A correct match between a generated derivative and the true suspect identity is defined as an exact match between the first 14 characters of the InChiKeys, which represent a two-dimensional structure.

### Ranking Candidate Structures

The final step involves ranking the generated candidate structures. Multiple candidates can be generated for each anchor–suspect pair because biotransformation rules might be applied to different atoms in the anchor molecule or because multiple rules match the observed precursor mass difference. Therefore, a systematic approach is needed to prioritize the suggested chemical identities of the suspect. For this, we use GNN-SOM,^12^ a tool that employs a graph neural network to predict the likelihood of each atom in a molecule being the target of a biotransformation operation. GNN-SOM was trained on enzymatic interactions from the KEGG database to classify each atom within a graph representation of a molecule as the site-of-metabolism for a given enzyme. BAM uses GNN-SOM to rank the generated derivatives based on the likelihood of their corresponding site-of-metabolism, with each derivative being assigned the likelihood of the specific atom to which the biotransformation rule was applied.

The quality of ranking is assessed by determining the rank of the true suspect among all candidates. To resolve ties, the average rank is computed by summing all ranks, including duplicates, and dividing by the number of tied candidates. For example, if three candidates are tied for first place, the average rank would be calculated as 2, and all three candidates would be assigned this rank. Additionally, we report the proportion of true suspect identities ranked at position *k*, where *k* ranges from 1 to 5. As GNN-SOM is the only learned step of our method, and it was previously pretrained on KEGG rather than RetroRules, no additional training was performed in the context of BAM. Thus, all suspect compounds in the anchor–suspect pairs that were derived from the global molecular network are treated as unknowns when applying BAM, and our data set acts as a test set for our evaluation.

### Comparative Analysis against SIRIUS

To evaluate the performance of BAM, we applied SIRIUS (version 5.6.3)^22^ to the query spectra represented in the same data set of 60,368 anchor–suspect pairs. SIRIUS is a computational tool that, given a query spectrum, generates fragmentation trees and predicts fingerprints that can then be compared to a molecular structure database for annotation. In this case, we used the command line interface to apply SIRIUS and the biomolecule structure database (bioDB) that was released with SIRIUS, which contains molecules of biological interest compiled from over 20 different data resources.^22^ To reduce computational run time, we first performed molecular formula annotation, and then only if the correct formula was generated, we performed molecular structure annotation. SIRIUS settings included adduct, ionization mode, and instrument type of spectra as specified by the corresponding metadata in GNPS/MassIVE, with all remaining settings left at their default values, including the default elements (C, H, N, O, P, S, Br, Cl, B, Se) and mass accuracy (10 ppm). When filtering for spectra that represent molecular structures present in bioDB, there are 38,368 unique query spectra in our data set representing 60,368 anchor–suspect pairs, because one anchor may have multiple suspect relationships and vice versa. Thus, we applied SIRIUS to each of the 38 368 spectra corresponding to a unique compound present in bioDB and evaluated it based on whether it was able to correctly generate the matching candidate structure as annotated through library searching while performing GNPS molecular networking.^1^ All code, data, and results relevant to this analysis can be found on Zenodo (DOI:10.5281/zenodo.13903946).

### Software Availability

BAM was implemented using two different code environments. First, PROXIMAL2 was applied and its results were analyzed using Python 3.9, PubChemPy (version 1.0.4),^23^ BioServices (version 1.11.2),^24^ scikit-learn (version 1.1.2),^25^ NumPy (version 1.23.2),^26^ NetworkX (version 2.5),^27^ RDKit (version 2022.03.5),^28^ KCF-Convoy (version 0.0.5),^29,30^ Pandas (version 1.4.3),^31,32^ Matplotlib (version 3.5.3),^33^ and Seaborn (version 0.12.2).^34^ Second, GNN-SOM was applied using Python 3.10, RDKit (version 2022.03.5),^28^ PyTorch (version 1.12.1),^35,36^ and PyTorch Geometric (version 2.1.0).^37^ All code, examples files, and detailed instructions are available as open source under the MIT license at https://github.com/HassounLab/BAM.

## Results and Discussion

**Figure 2:**
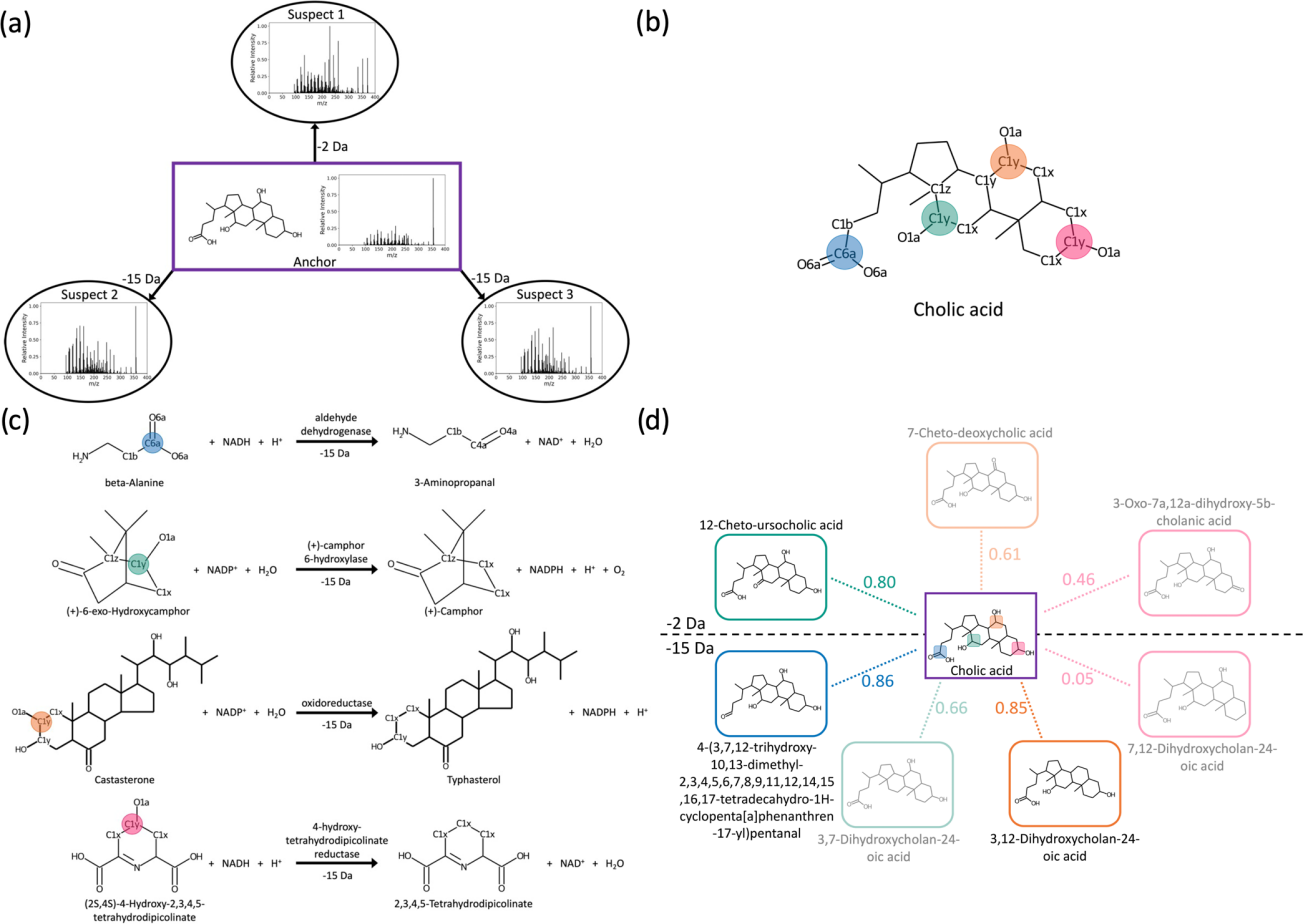
Demonstration of BAM using cholic acid as an anchor molecule to annotate three query spectra. (a) The anchor molecule, cholic acid, has high spectral similarity with three unidentified suspects. The precursor *m*/*z* differences between the anchor and suspects spectra correspond to −15 and −2 Da. (b) Biotransformation rules are selected by the observed mass differences and are only applied if the corresponding reaction center is exhibited in the anchor structure. Selected biotransformations rules are applied to four different reaction centers, which are highlighted by different colors here. The KEGG atom type of the reaction centers as well as the neighboring atoms are displayed as well. (c) The four relevant biotransformation rules that exhibit a mass difference of −15 Da are identified. Each biotransformation rule may be applied to the corresponding reaction center of cholic acid, as indicated by color. (d) Application of BAM: The four depicted biotransformation rules are applied to cholic acid to generate four derivative candidates for suspect 2 and 3 that have a mass difference of −15 Da. Three other biotransformation rules are applied to cholic acid to generate three derivative candidates for suspect 1 that have a mass difference of −2 Da. The generated derivative candidates are ranked based on the predicted likelihood of each atom within the anchor molecule serving as a reaction center. The figure distinguishes candidates derived from the −2 Da rules (above the dashed line) and those from the −15 Da rules (below the dashed line). The likelihood of each derivative structure being the correct annotation is indicated along corresponding colored dotted lines. The true structures of the three suspects are the top-ranked candidates (displayed without shading). The top-ranked candidate for the −2 Da mass difference—12-Cheto-ursocholic acid—matches the actual structure of suspect 1. Similarly, the two highest-ranked candidates for the −15 Da mass difference—4-(3,7,12-trihydroxy-10,13-dimethyl-2,3,4,5,6,7,8,9,11,12,14,15,16,17-tetradecahydro-1*H*-cyclopenta[*a*]phenanthren-17-yl)pentanal and 3,12-Dihydroxycholan-24-oic acid—both displaying significant structural similarity, match the true structures of the remaining two suspects.

An example application of BAM on three query spectra representing three unique suspects is demonstrated (Figure 2). All query spectra exhibit high spectral similarity to the same spectrum associated with the same anchor, cholic acid (Figure 2a). BAM identifies relevant mass differences between the suspects and the anchor, selects appropriate biotransformation rules corresponding to these mass differences (Figure 2b), and applies them to generate and rank multiple derivative candidates (Figure 2c). In this case, BAM was able to successfully identify the true structures of the suspects, showcasing its potential in discovering new molecules corresponding to previously unknown MS/MS spectra. Consequently, BAM not only enhances the accuracy of compound identification from untargeted metabolomics data, but it also enables the discovery of novel molecules, thereby enriching our comprehension of metabolic processes and their vast array of products.

Next, we applied BAM to data from the global molecular network that was recently compiled from hundreds of millions of public MS/MS spectra.^15^ To determine a data set of known suspects to evaluate BAM, a set of 30,184 structurally unique, annotated spectrum pairs were derived from neighboring annotated nodes from this global molecular network, yielding 60,368 total anchor–suspect pairs. As biotransformations are reversible, each such pair yields two possible anchor–suspect pairs, with each compound within the pairs in turn serving as an anchor while the other serves as a suspect. Thus, when querying these 60,368 suspects in step 1 of BAM, the corresponding anchors are determined as neighboring nodes to the suspects in the global molecular network.

Additionally, we utilized RetroRules^9^ as the basis for extracting biotransformation rules—yielding 42,307 unique rules—covering a wide range of possible biochemical transformations. These rules were extracted from the 351,704 biotransformation rules in RetroRules, which represent 84,614 reactant–product pairs, using PROXIMAL2. When rounded to unit resolution, observed precursor mass differences matched 17,254 biotransformation rules, corresponding to 41% of rules from the RetroRules database that are applicable for BAM. Conversely, 60,320 out of 60,368 anchor–suspect pairs exhibit a mass difference corresponding to at least one biotransformation rule, indicating that almost all queries could potentially be explained by a rule from the RetroRules database. The remaining 48 anchor–suspects pairs that cannot be explained by these rules can be attributed to either an unknown biotransformation or a non-biochemical relationship between the anchor and suspect molecules.

**Figure 3:**
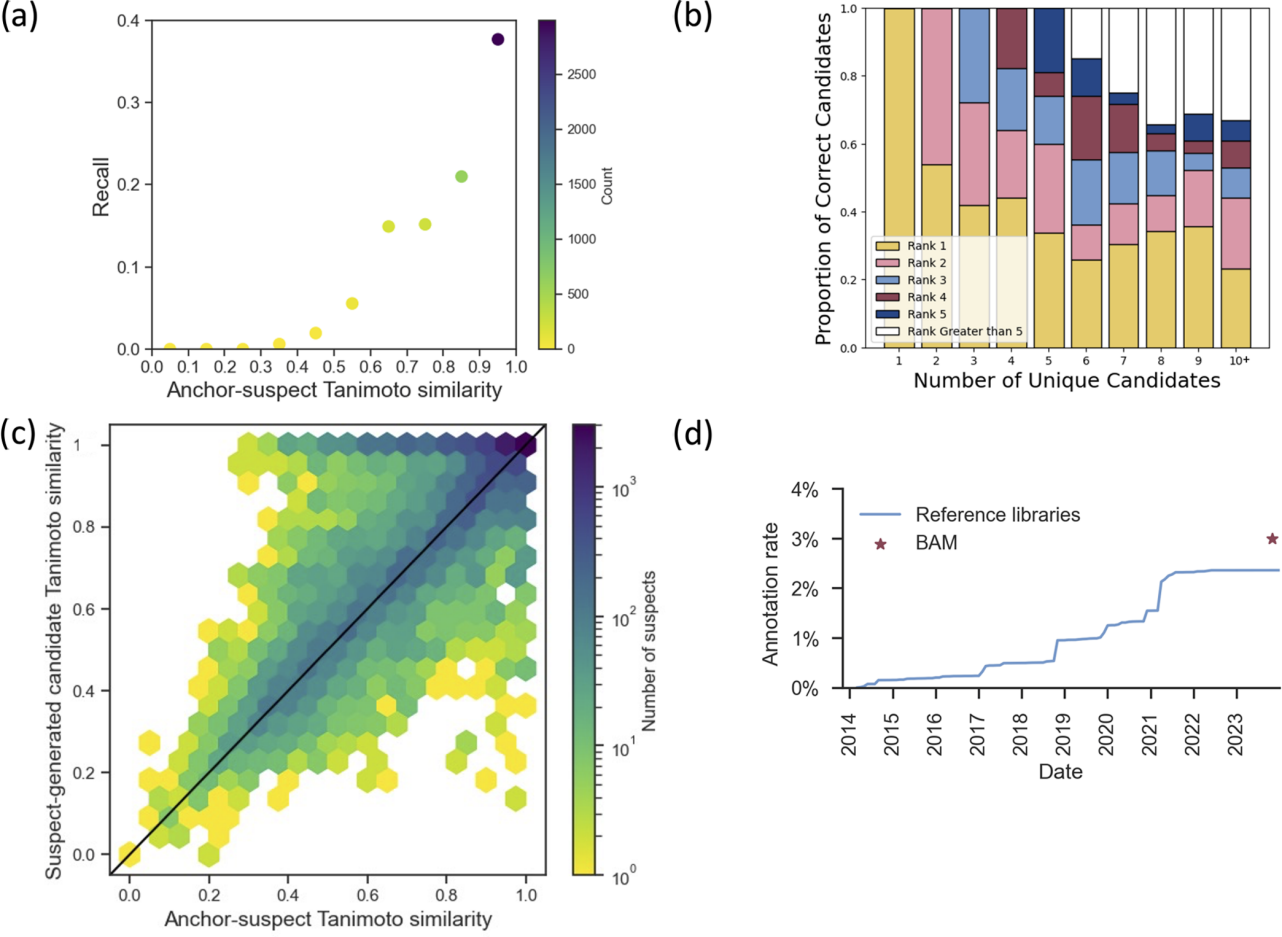
Performance evaluation of BAM. (a) Recall of BAM as a function of the structural similarity between the anchor and suspect molecules. The recall and the count capture the proportion and the number of queries for which BAM generated the true structure when a derivative is generated, respectively. The count is represented by the color of each point. (b) Rank of the true structure of the suspect for instances in which derivatives could be generated. (c) Anchor–suspect Tanimoto similarity versus suspect–generated candidate Tanimoto similarity. In 74.0% of cases (observations on or above the diagonal), BAM generated a candidate more similar to the suspect than the anchor. The BAM candidates therefore mark an improvement in structural hypotheses for metabolite annotation of previously unknown MS/MS spectra. (d) Contribution offered by BAM in the context of the historical increase in MS/MS spectrum annotation rates,^2^ as determined by spectral library searching performed as part of the systematic living data reanalysis on GNPS.^1^.

Applying BAM resulted in 17,271 queries generating one or more derivatives, and the average number of unique derivatives generated is 3.65 (±4.29). Among these, BAM successfully generated the correct suspect structure in 4171 cases, achieving a recall of 24.2% when a derivative is generated. Notably, when the structural similarity between the anchor and suspect was restricted to above 0.9 Tanimoto similarity, the recall increased significantly to 37.7%, highlighting BAM’s effectiveness for similar molecules (Figure 3a). Furthermore, BAM demonstrated impressive accuracy in ranking the correct derivatives, achieving an average rank of 2.1 and ranking the correct suspect first in 57.7% of cases (Figure 3b).

An analysis of the derivatives generated by BAM revealed that in 74.0% of cases, the candidates were more similar or equally similar to the suspect than to the anchor molecule (Figure 3c). This indicates that BAM can not only identify potential suspect structures, but also improves upon structural hypotheses for metabolite annotation than standard molecular networking or analog searching. Such a high degree of similarity enhancement is a testament to BAM’s ability to accurately interpret and utilize biochemical transformations for MS/MS spectrum annotation.

We additionally performed a comparative analysis against SIRIUS. SIRIUS generated the true molecular formula in 4756 cases and the true molecular structure in 4560 cases out of 38,368 molecular structures available in bioDB. As both BAM and SIRIUS provide structural ranking, we report the number of queries that generated and ranked the true structure within top *k* ranks, where *k* = 1, 3, 5, 10, and with all ranks considered (Table 1). Because BAM and SIRIUS both generate a variable number of candidates for each query, we only included cases where at least *k* structures are generated. Importantly, when reporting the number of correct queries that are unique to SIRIUS and to BAM, the majority of correct queries are unique to each method. This observation confirms the value of utilizing biotransformation rules for structural elucidation, and demonstrates that the two tools provide complementary annotation results.

**Table 1:** Comparative Results of SIRIUS and BAM Evaluated on a Dataset of 38,368 Unique Queried Spectra, Where the Target Molecule is in bioDB^a^

*k*	SIRIUS	BAM	unique to SIRIUS	unique to BAM
1	2758	1478	2499	1219
3	3575	1014	3383	822
5	3703	684	3596	577
10	3718	294	3696	272
all ranks considered	4560	3719	3830	2989

a The first two columns represent the number of queries that generated and ranked the true structure at *k* or higher by SIRIUS and BAM, when restricting to cases where the number of generated candidates is greater than *k*. The last two columns represent the number of those correct queries that were uniquely generated by SIRIUS and BAM. The bottom row reports the results irrespective of rank.

For a more detailed analysis regarding the top k rank against the number of candidates, we provide the number of queries that generated and ranked the true structure within the top *k* ranks, when at least n candidates were generated, for *k* = 1, 3, 5, 10 and *n* = 1, 3, 5, 10 (Table 2). Consequently, the numbers in the diagonals of Table 2A,B are the same as the first two columns of Table 1 where *k* = *n*. The first column of Table 2, where *n* = 1, represents the number of queries that generated the true structure within the top *k* candidates where at least one candidate was generated. At all ranks, SIRIUS generated a higher number of correct structures than BAM. Additionally, the number of queries that generated the true structure within *k* rank by SIRIUS (Table 2A) decreases less substantially as the number of generated candidates increases than those same results by BAM (Table 2B). These results are expected, as BAM explores biotransformation rules while SIRIUS is a more general technique that utilizes fragmentation trees and machine learning for structural elucidation, and BAM often generates less candidates on average than SIRIUS.

**Table 2:** Number of Cases Where the True Structure was Generated and Ranked Within the Top *k* candidates (rows), When at Least *n* number of Candidates were Generated (columns)^a^

*k*/*n*	1	3	5	10
(A) SIRIUS				
1	2758	2718	2620	2407
3	3623	3575	3466	3203
5	3862	3814	3703	3437
10	4143	4095	3984	3718
(B) BAM				
1	1478	263	105	22
3	2751	1014	373	83
5	3213	1476	684	113
10	3563	1826	1034	294

a For example, the correct structure was at rank *k* = 3 or higher in 373 cases by BAM where at least *n* = 5 candidates were generated.

When considering the increase in spectrum annotation rate afforded by BAM, its potential becomes even more apparent. As each node in the global molecular network represents a number of experimental spectra, the 4171 correctly annotated queries map to 219,985 spectra across 466 GNPS/MassIVE data sets. The BAM approach is therefore widely applicable and can potentially provide novel insights for a diverse range of biological studies, underscoring its broad relevance and effectiveness. Extrapolating the recall rate from these findings to all anchor–suspect pairs in the global molecular network, BAM could annotate approximately 1,135,000 previously unknown MS/MS spectra from 1129 data sets in GNPS/MassIVE, which increases the overall annotation rate from 2.3 to 3% (Figure 3d). This substantial enhancement in annotation capabilities marks a significant leap forward in metabolite discovery and understanding, as BAM not only boosts the recall of correct structural assignments but also offers richer structural hypotheses for previously unannotated metabolites.

Looking ahead, there are opportunities to further refine and enhance BAM’s performance. One promising avenue is the extraction of additional biotransformation rules from specific biochemical reactions, particularly those most relevant to the biological samples under study. BAM performance may be further improved by considering multistep biotransformations that yield the observed mass difference between the anchor and suspect. While GNN-SOM was trained on enzymatic reactions in the KEGG database, which contain reactions for over 8000 species, we anticipate improvements in ranking accuracy of BAM to be achieved by fine-tuning GNN-SOM using the more extensive data from RetroRules. Furthermore, the integration of MS/MS data during scoring, along with the consideration of multiple annotated spectra representing different anchors, could improve the generation and ranking of candidate structures. For instance, candidates for a suspect derived from different anchors could be ranked based on a consensus metric, providing a more robust and accurate annotation.

In conclusion, BAM’s application to the global molecular network signals an important step forward in metabolomics, enabling discovery and annotation of novel analog molecules in a data-driven fashion, rather than being restricted by exact matching to existing spectral libraries with limited coverage. BAM’s methodology, which harnesses vast public MS/MS data and biotransformation rules, paves the way for deeper insights into metabolic processes and significantly enriches the field of metabolite annotation. With ongoing enhancements and refinements, BAM is poised to unlock even more potential in the exploration and understanding of the complex world of metabolomics.
